# Remifentanil-Based Monitored Anesthesia Care in a Paraplegic Patient With Severe Kyphoscoliosis, Non-palpable Spinal Landmarks, and Coagulation Abnormality Undergoing Ischiorectal Abscess Drainage: A Case Report

**DOI:** 10.7759/cureus.105410

**Published:** 2026-03-17

**Authors:** Rahul Kalshan, Shilpa Bajaj, Dhirja Sharma, Trisha Nidhi, Ranju Gandhi

**Affiliations:** 1 Anesthesiology, Holy Family Hospital, New Delhi, IND; 2 Anesthesia, Vardhman Mahavir Medical College (VMMC) and Safdarjung Hospital, New Delhi, IND; 3 Anesthesia and Intensive Care, Vardhman Mahavir Medical College (VMMC) and Safdarjung Hospital, New Delhi, IND

**Keywords:** difficult airway, gibbus deformity, monitored anesthesia care, neuraxial anesthesia limitation, paraplegia, remifentanil infusion, severe kyphoscoliosis

## Abstract

Severe kyphoscoliosis with prior spinal surgery presents significant anesthetic challenges, including anticipated difficult airway, restrictive pulmonary physiology, and technical impracticality of neuraxial anesthesia. We report the anesthetic management of a 23-year-old paraplegic female patient with a massive dorsal gibbus deformity and completely non-palpable spinal landmarks who presented with recurrent ischiorectal abscess. Preoperative evaluation revealed moderate anemia, leukocytosis, mild hypokalemia, and prolonged international normalized ratio (INR). Due to inability to tolerate the supine position, anticipated airway difficulty, unknown prior spinal instrumentation, and relative contraindication to neuraxial anesthesia, the procedure was performed in the left lateral position under remifentanil-based monitored anesthesia care with adjunct ketamine and midazolam while maintaining spontaneous ventilation. Surgery was completed successfully without airway instrumentation, hemodynamic instability, or arrhythmias. This case highlights individualized anesthetic planning and the role of remifentanil infusion as a primary technique in high-risk patients where both general and spinal anesthesia pose significant challenges.

## Introduction

Kyphoscoliosis is characterized by combined coronal and sagittal spinal deformity and is associated with restrictive lung disease, impaired chest wall compliance, and increased perioperative respiratory risk [1,2]. When Cobb's angle exceeds 50°, progressive cardiopulmonary compromise becomes clinically significant [2].

Anesthetic management in such patients is challenging due to an anticipated difficult airway, reduced pulmonary reserve, positioning limitations, and potential difficulty with neuraxial techniques [3]. Prior spinal surgery further complicates regional anesthesia because of altered anatomy, fibrosis, and unpredictable local anesthetic spread [4].

Remifentanil, an ultra-short-acting μ-opioid receptor agonist, has a rapid onset and context-sensitive half-life of approximately 3-5 minutes, allowing precise titration and rapid recovery [5]. When carefully titrated, remifentanil allows the preservation of spontaneous ventilation and has been used effectively in monitored anesthesia care [6].

We describe the successful use of remifentanil-based sedation as the primary anesthetic technique in a patient where both general and neuraxial anesthesia were considered high risk.

## Case presentation

**Figure 1:**
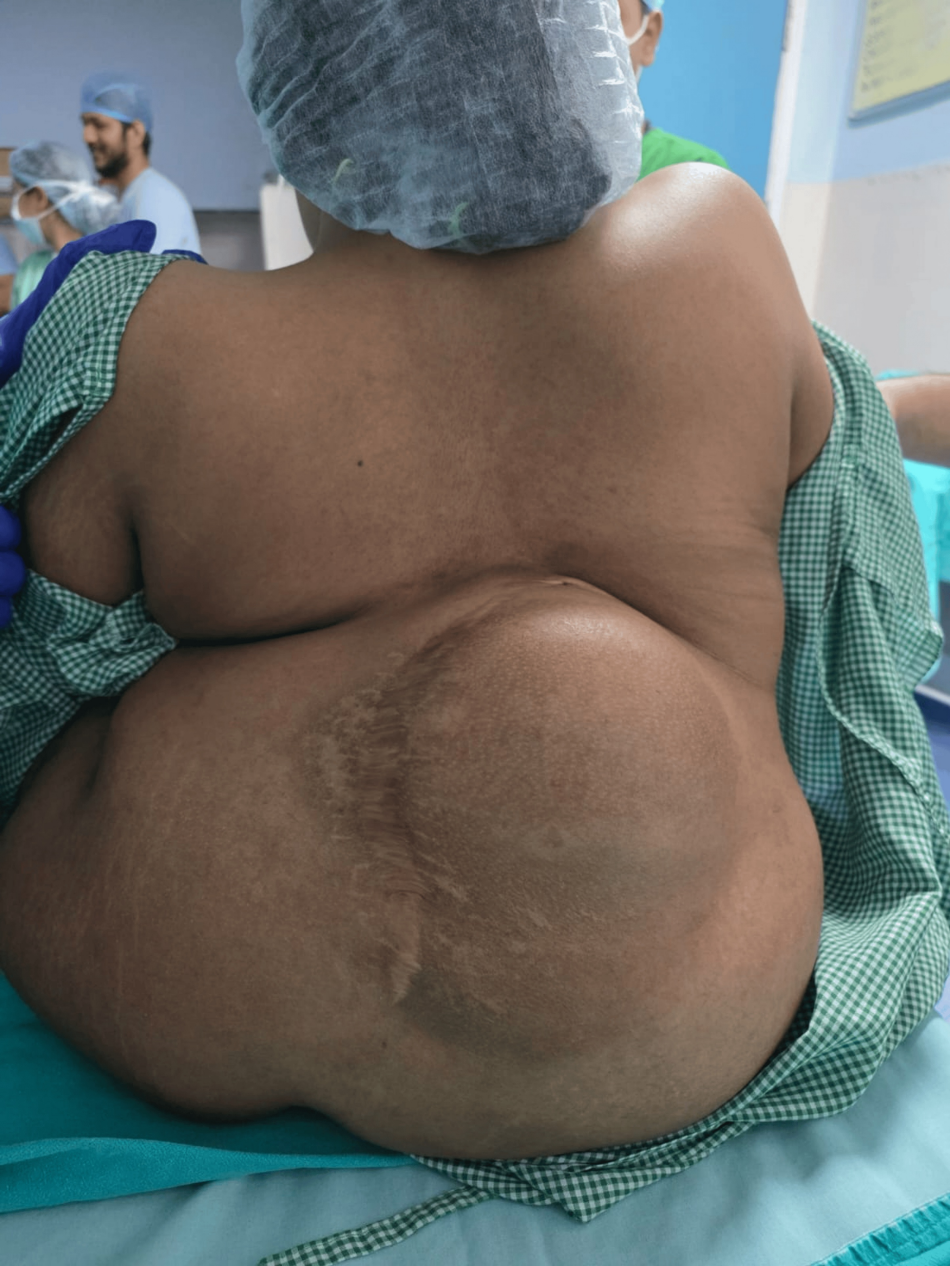
Clinical photograph demonstrating massive dorsal gibbus deformity with severe kyphoscoliosis and distorted spinal anatomy

A 23-year-old female patient with congenital paraplegia residing in an orphanage presented to Holy Family Hospital, New Delhi, India, with a recurrent ischiorectal abscess requiring incision and drainage. She had undergone spinal surgery during childhood; however, operative records were unavailable. Clinical examination revealed a massive dorsal gibbus deformity with severe kyphoscoliosis (Cobb's angle >50°), resulting in gross distortion of the paraspinal anatomy with completely non-palpable spinous processes (Figure 1). The patient was unable to tolerate the supine position due to the spinal deformity.

Airway evaluation was limited because of positioning intolerance, raising concern for potential difficult airway management if general anesthesia were required. Baseline oxygen saturation was 98-100% on room air. Transthoracic echocardiography demonstrated normal cardiac function.

Preoperative laboratory investigations are summarized in Table 1. Despite mild hypokalemia (3.09 mmol/L), surgery proceeded given the minor nature of the procedure, absence of planned neuromuscular blockade, and continuous electrocardiographic monitoring.

**Table 1:** Preoperative laboratory investigations These findings indicated moderate anemia, active infection, mild hyponatremia, hypokalemia, and mildly prolonged coagulation parameters. INR: international normalized ratio

Investigation	Result	Reference range
Hemoglobin	8 g/dL	12-16 g/dL
Total leukocyte count	19,600/mm³	4,000-11,000/mm³
Platelet count	4.91×10⁵/mm³	1.5-4.5×10⁵/mm³
Blood urea	12 mg/dL	7-20 mg/dL
Serum creatinine	0.2 mg/dL	0.6-1.2 mg/dL
Sodium	134 mmol/L	135-145 mmol/L
Potassium	3.09 mmol/L	3.5-5.0 mmol/L
Chloride	102 mmol/L	98-106 mmol/L
Bicarbonate	21 mmol/L	22-28 mmol/L
Prothrombin time	17.6 sec	11-13.5 sec
INR	1.51	0.8-1.2

**Figure 2:**
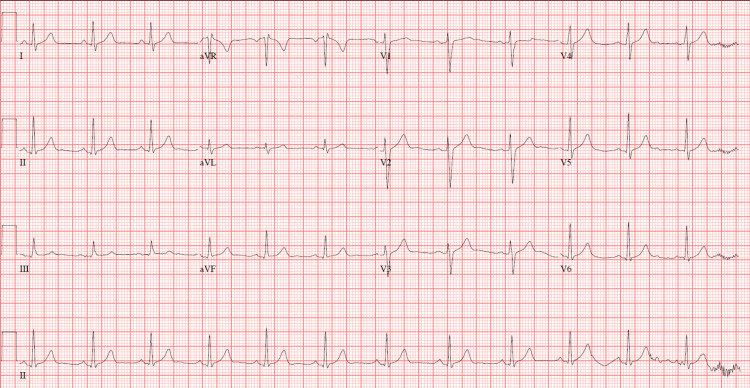
Baseline electrocardiogram showing normal sinus rhythm without ST-T abnormalities

Baseline electrocardiography showed a normal sinus rhythm without conduction abnormalities (Figure 2). Neuraxial anesthesia was considered technically impractical due to severe spinal deformity, completely non-palpable landmarks, unknown prior spinal instrumentation, and an international normalized ratio (INR) of 1.51 representing a relative contraindication. General anesthesia was also considered high risk because of an anticipated difficult airway, inability to position the patient supine, and restrictive lung mechanics associated with severe kyphoscoliosis.

After a multidisciplinary discussion, the decision was made to proceed with monitored anesthesia care in the left lateral position. A contingency plan for local anesthetic infiltration by the surgical team was kept available if required.

Standard American Society of Anesthesiologists (ASA) monitoring, including electrocardiography, non-invasive blood pressure monitoring, pulse oximetry, and respiratory rate monitoring, was instituted. Oxygen was administered via face mask at 6 L/min. Sedation was initiated with remifentanil 0.5 µg/kg intravenous bolus followed by continuous infusion at 0.2 µg/kg/min. Adjunct medications included ketamine 10 mg intravenous and midazolam 1 mg intravenous. Spontaneous ventilation was maintained throughout the procedure. Hemodynamic parameters remained stable, and no episodes of oxygen desaturation or arrhythmias were observed. The surgery was completed successfully in the lateral position without airway instrumentation, and postoperative recovery was uneventful.

## Discussion

This case illustrates complex anesthetic decision-making in a patient with severe spinal deformity and systemic derangements.

Airway and respiratory considerations

Severe kyphoscoliosis reduces functional residual capacity and pulmonary compliance, predisposing to hypoventilation and hypoxemia under general anesthesia [1,2]. Difficult airway risk increases due to limited neck mobility and altered anatomy [3]. Avoiding general anesthesia eliminated the need for airway instrumentation and positive pressure ventilation.

Neuraxial anesthesia challenges

Previous spinal surgery and severe deformity significantly increase failure rates and technical difficulty of neuraxial blocks [4,7]. Completely non-palpable landmarks and altered vertebral anatomy increase the risk of traumatic insertion and unpredictable block height. Additionally, an INR of 1.51 represented a relative contraindication to neuraxial anesthesia due to bleeding risk [8].

Laboratory abnormalities

Moderate anemia reduced physiologic reserve. Leukocytosis reflected active infection. Mild hypokalemia (3.09 mmol/L), although not corrected preoperatively, was considered acceptable given the absence of muscle relaxants and continuous electrocardiogram (ECG) monitoring. No perioperative arrhythmias occurred.

Role of remifentanil

Remifentanil's rapid titratability, short context-sensitive half-life, and minimal accumulation make it particularly useful for monitored anesthesia care [5,6]. When combined with low-dose ketamine, adequate analgesia is achieved while preserving respiratory drive [9]. This approach provided stable hemodynamics and excellent intraoperative analgesia without airway manipulation.

The case underscores the importance of individualized planning, especially in socially vulnerable patients with incomplete medical records.

## Conclusions

Severe kyphoscoliosis with massive dorsal gibbus and non-palpable spinal landmarks presents profound anesthetic challenges. When both general and neuraxial anesthesia pose significant risks, remifentanil-based monitored anesthesia care may serve as a safe and effective alternative. Meticulous preparation, vigilant monitoring, and a predefined backup strategy are essential to achieving favorable outcomes in such complex cases.
